# Autoimmune Hepatitis with Concomitant Idiopathic Thrombocytopenic Purpura Diagnosed by Transjugular Liver Biopsy

**DOI:** 10.1155/2018/5305691

**Published:** 2018-12-09

**Authors:** Hiromi Fukuda, Kazuhide Takata, Takanori Kitaguchi, Ryo Yamauchi, Hideo Kunimoto, Takashi Tanaka, Keiji Yokoyama, Daisuke Morihara, Yasuaki Takeyama, Satoshi Shakado, Ai Mogi, Shinichi Kora, Kaori Koga, Morishige Takeshita, Kengo Yoshimitsu, Shotaro Sakisaka

**Affiliations:** ^1^Department of Gastroenterology and Medicine, Fukuoka University Faculty of Medicine, 7-45-1 Nanakuma, Jonan, Fukuoka 814-0180, Japan; ^2^Division of Medical Oncology, Hematology and Infectious Disease, Department of Internal Medicine, Fukuoka University Faculty of Medicine, 7-45-1 Nanakuma, Jonan, Fukuoka 814-0180, Japan; ^3^Department of Radiology, Fukuoka University Faculty of Medicine, 7-45-1 Nanakuma, Jonan, Fukuoka 814-0180, Japan; ^4^Department of Pathology, Fukuoka University Faculty of Medicine, 7-45-1 Nanakuma, Jonan, Fukuoka 814-0180, Japan

## Abstract

Patients with autoimmune hepatitis (AIH) may sometimes have concomitant idiopathic thrombocytopenic purpura (ITP). Severe thrombocytopenia in ITP interferes with percutaneous liver biopsy for pathological diagnosis of AIH. Here, we report a case of AIH with ITP in a 63-year-old woman. The patient presented to our hospital with liver dysfunction and thrombocytopenia. For histological examination, transjugular liver biopsy (TJLB) was performed, leading to a diagnosis of AIH. Corticosteroids treatment led to an improvement in her liver enzyme levels and platelet count. In conclusion, patients with AIH may sometimes have concomitant ITP. TJLB was effective for making the diagnosis of AIH with severe thrombocytopenia due to ITP.

## 1. Introduction

Autoimmune hepatitis (AIH) is characterized by chronic inflammation of the liver, hypergammaglobulinemia and production of autoantibodies, and a favorable response to immunosuppressive therapy. Histological examination, which tends to reveal interface hepatitis and plasma cell infiltration, is important for the diagnosis of AIH [1]. AIH can often occur in the setting of other nonhepatic autoimmune disorders, such as chronic thyroiditis (7.5%) and Sjögren's syndrome (5.7%); however, concomitant AIH and idiopathic thrombocytopenic purpura (ITP) is rare (0.6%) [2].

Percutaneous liver biopsy (PLB) is the standard method for obtaining a liver procuring tissue for histological examination. When it is contraindicated because of the risk of postprocedure hemorrhage, transjugular liver biopsy (TJLB) is recommended.

Here, we report a case of AIH associated with ITP, in which TJLB was useful in making the diagnosis and prompts start of treatment with a corticosteroid.

## 2. Case Presentation

A 63-year-old woman with no significant medical history was referred to our hospital for further investigation of elevated liver enzyme levels and thrombocytopenia. The patient had a few days' history of general malaise and purpura of her legs. She had no fever or any abdominal complaints. She had a history of alcohol intake of about 40 g/day and no constant drug use.

Her vital signs were stable and physical findings were normal except for jaundice and purpura of her legs. The laboratory findings were as follows: total bilirubin, 8.8 mg/dL; aspartate aminotransferase (AST), 1,767 U/L; alanine aminotransferase (ALT), 1,845 U/L; *γ*-glutamyl transpeptidase, 2,229 U/L; alkaline phosphatase (ALP), 845 U/L; immunoglobulin G (IgG), 2,042 mg/dl; anti-nuclear antibody (ANA) titer, positive at 80-fold dilution; platelet count, 22,000/*μ*L; platelet-associated IgG (PAIgG), 208 ng/10^∧^7 cells. Serologic markers for hepatitis A, B, C, and E viruses, and Epstein-Barr virus, cytomegalovirus, varicella zoster virus, and herpes simplex virus were all negative, and anti*-Helicobacter pylori* (*H. pylori*) IgG was positive (Table 1). Abdominal ultrasonography and enhanced computed tomography revealed no significant biliary tract disease that could have led to liver damage.

According to the criteria of the International Autoimmune Hepatitis Scoring System, the patient's pretreatment clinical score without histology was 13 (female: +2; ALP/ALT ratio: +2; IgG level: +1; ANA titer: +2, antimitochondrial antibody: 0; viral markers: +3; drugs: +1; alcohol: 0; immune disease: +2), indicating probable AIH. Her severe thrombocytopenia was considered to be due to concomitant ITP because of her clinical and laboratory findings.

**Figure 1:**
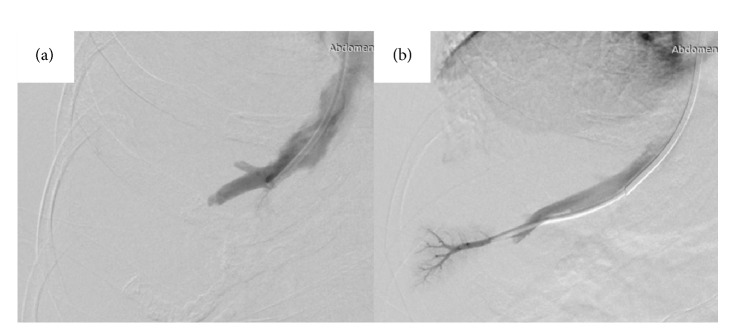
Transjugular liver biopsy was performed under X-ray fluoroscopy guidance. (a) A venogram showing a 5-Fr catheter inserted into the right hepatic vein. (b) A postbiopsy venogram, showing no leakage of contrast agent to the outside of the liver.

Liver biopsy via the transjugular route (TJLB) was selected to confirm the diagnosis of AIH to avoid the risk of hemorrhage after percutaneous liver biopsy (PLB) in a patient with severe thrombocytopenia. The right internal jugular vein was punctured after administering local anesthesia, and a 5-Fr catheter cannulated into the right hepatic vein (RHV) over the guide wire. A venogram was performed to confirm the position of the catheter in the RHV (Figure 1(a)). The catheter was then exchanged for a transjugular liver access and biopsy set catheter (Cook Medical, Bloomington, IN). Five liver biopsy specimens were obtained. All steps were performed using X-ray fluoroscopy to confirm the location of the guide wire or catheter. A postbiopsy venogram revealed no complications, including intraperitoneal hemorrhage (Figure 1(b)). Manual compression of the access site on her neck was applied from catheter removal until hemostasis was achieved.

**Figure 2:**
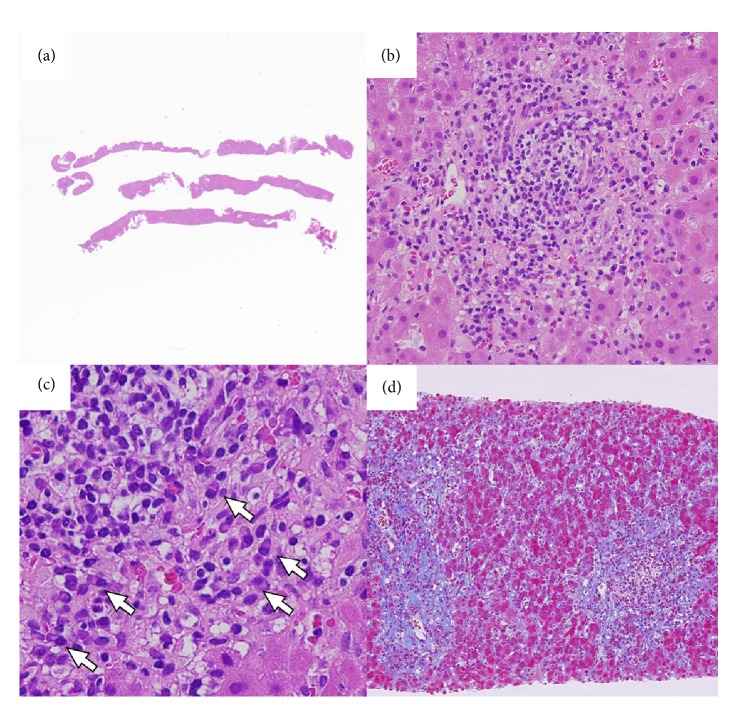
The histological findings of liver biopsy. (a) The specimens of transjugular liver biopsy. (b, c) Interface hepatitis with moderate inflammation and infiltrates of lymphocytes and plasma cells (arrows) in the portal area (b, magnification: ×200. Hematoxylin and eosin staining; c, magnification: ×400; hematoxylin and eosin staining). (d) Moderate fibrosis in the portal area and partial destruction of the hepatic lobules with piecemeal necrosis (magnification: ×40; Masson's trichrome staining).

**Figure 3:**
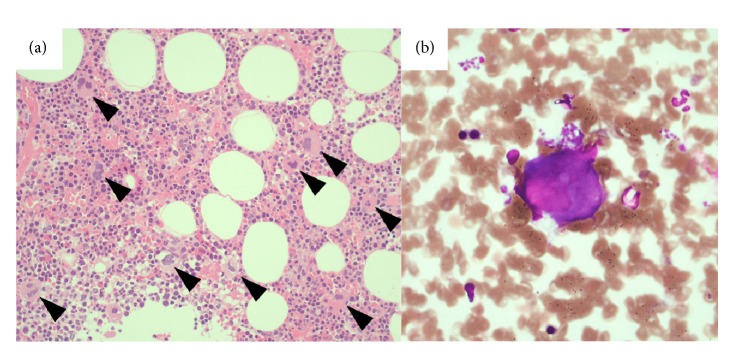
The histological findings of bone marrow biopsy and aspiration. (a) The bone marrow was normocellular with increased megakaryocytes (arrow heads) (magnification: ×200; hematoxylin and eosin staining). (b) Few platelets were visualized around and near the megakaryocytes (magnification: ×400; Wright-Giemsa staining).

**Figure 4:**
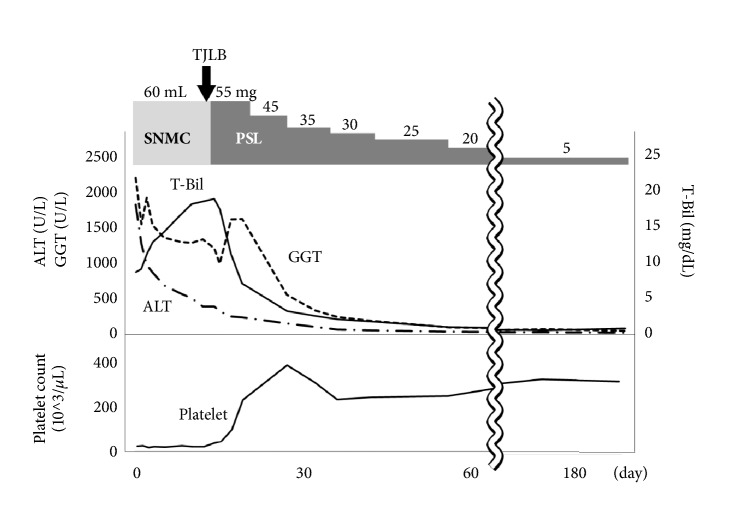
Clinical course. T-Bil: total bilirubin; ALT: alanine aminotransferase; GGT: gamma-glutamyl transferase; TJLB: transjugular liver biopsy; SNMC: Stronger Neo-Minophagen C; PSL: prednisolone.

The histological findings of liver biopsy showed interface hepatitis and moderate to severe inflammatory infiltrates including lymphocytes and plasma cells. Moderate fibrosis in the portal area and partial destruction of the hepatic lobules with partial piecemeal necrosis was also observed (Figures 2(a), 2(b), 2(c), and 2(d)). All these pathological findings were compatible with the diagnosis of AIH, and the postbiopsy score was 17, thereby confirming the diagnosis of AIH. Bone marrow aspiration revealed a normal nucleated cell count and a slight increase in megakaryocytes, suggestive of ITP (Figure 3). She received an intravenous glycyrrhizin-containing herbal medicine, Stronger Neo-Minophagen C (SNMC; Minophagen Pharmaceutical, Tokyo, Japan) at 60 ml/day before TJLB, and prednisolone (PSL) at 55 mg/day (0.8 mg/kg/day) after the histological diagnosis of AIH. After PSL administration, the patient demonstrated a good response with restored liver enzyme levels and platelet counts (Figure 4). PSL was tapered to 5 mg/day as a maintenance dose. The patient recovered uneventfully except for a hyperglycemic event requiring insulin treatment.

## 3. Discussion

The present case demonstrates two important clinical points: patients with AIH may sometimes have concomitant ITP, and TJLB is a valuable modality for the diagnosis of AIH with ITP.

With regards to the first point, both AIH and ITP are autoimmune-mediated disorders. AIH is defined as a chronic progressive liver disease caused by unknown etiological factors that is associated with aberrant autoreactivity and a genetic predisposition. In contrast, ITP is caused by immune system dysregulation and the development of autoantibodies for platelet surface glycoproteins (GP) including GPIIb-IIIa, GPIb-IX, and GPIa-II [3, 4]. The prevalence of ITP among patients with AIH in Japan is reported to be 0.6% [2], and there are numerous previous case reports of AIH associated with ITP [5–12]. Although the frequency of autoantibodies against platelet surface GP in patients with AIH remains poorly understood [7–9], some previous reports described that HLA class II genes, which are associated with susceptibility to AIH, influence the production of anti-GP antibody specificities, suggesting a relationship between AIH and ITP [13, 14].

The presence of autoimmune diseases other than ITP should be noted on clinical practice. Although not admitted in this case, there are numerous previous reports of AIH complicated with other autoimmune diseases in addition to ITP, such as primary biliary cholangitis, Sjögren's syndrome, and chronic thyroiditis [2, 8–12].

Corticosteroids are the first-line therapy for both AIH and ITP, similar to several other autoimmune diseases. Our patient showed immediate improvement of liver function and elevation of platelet count after treatment with PSL.

Our report also showed how TJLB was effective for the diagnosis of AIH with severe thrombocytopenia due to ITP. As mentioned above, histological examination is important for the diagnosis of AIH. The most common approach to obtaining liver tissue is PLB, whose major possible complication is postprocedure hemorrhage. Therefore, it is contraindicated for patients with severe thrombocytopenia. TJLB has been most commonly performed in patients in whom the risks of conventional PLB were deemed unacceptable. In theory, bleeding into the extravascular or abdominal cavity does not occur during TJLB as the liver tissue is obtained through a cannula introduced into the hepatic vein using jugular venous access. Previous reports of TJLB have consistently reported complication rates of 1.3–6.5%, lower than those after PLB [15, 16]. With recent developments and improvements in devices, the quality of TJLB specimens has become comparable to those obtained with PLB [17, 18]. In our case, the liver specimen obtained after five punctures during TJLB was sufficient to make a histological diagnosis of AIH. Postprocedure angiography confirmed that bleeding and liver penetration did not occur. We elected to confirm the histological diagnosis by TJLB preceding treatment for the following two reasons: (i) confirming the histological diagnosis as soon as possible and (ii) avoiding overlooking other potential diagnoses, such as hepatic lymphoma, and preventing the effects of PSL on liver tissue, which may make histological diagnosis difficult. However, in hospitals wherein TJLB is unavailable, PLB might be considered after increasing the platelet count by corticosteroid administration. Previous reviews on* H. pylori* infection in patients with ITP have shown that eradication of* H. pylori* infection in patients with ITP results in increased platelet counts in about half of the cases; therefore, eradication treatment is recommended [19–21]. However, in this case, because there was a risk of exacerbation of liver damage, no eradication therapy was administered. Lusutrombopag, which is an oral thrombopoietin receptor agonist that reduces severe thrombocytopenia in patients with chronic liver disease who are scheduled to undergo an invasive procedure, may be a useful drug in this patient. [22]

The indications for TJLB include not only coagulation disorders, but also the presence of ascites, morbid obesity, previous liver transplant, acute liver failure, and patients in whom a PLB has failed [16].

In conclusion, patients with AIH may sometimes have concomitant ITP. TJLB was effective for making the diagnosis of AIH with severe thrombocytopenia due to ITP.

## Figures and Tables

**Table 1:** Laboratory data on admission.

**Hematology**		
White blood cell	5,100	/*μ*L
Red blood cell	482	10^∧^6/*μ*L
Hemoglobin	14.9	g/dL
Platelets	22	10^∧^3/*μ*L

**Coagulation**		
PT%	95	%
PT-INR	1.03	
APTT	26.1	

**Biochemistry**		
Albumin	3.9	g/dL
Total bilirubin	8.8	mg/dL
Direct bilirubin	6.0	mg/dL
AST	1,767	U/L
ALT	1,845	U/L
GGT	2,229	U/L
ALP	845	U/L
Amylase	39	U/L
BUN	10	mg/dL
Creatinine	0.7	mg/dL
eGFR	64.5	mL/min
CRP	0.8	mg/dL
TSH	0.363	*μ*IU/mL
FT4	1.59	ng/dL

**Immunology**		
IgG	2,042	mg/dL
IgM	105	mg/dL
IgG4	70	mg/dL
ANA	80	Dil
AMA (M2)	<1.5	Index
anti-LKM1 Ab	<5	Index
anti-thyroperoxidase Ab	10	IU/mL
anti-thyroglobulin Ab	11	IU/mL
anti-SSA Ab	<1.0	U/mL
anti-SSB Ab	<1.0	U/mL
PAIgG	208	ng/10^∧^7 cells (normal range ≦46)
HLA-DR	DR4, DR15	

**Infectious Makers**		
HCVAb	(-)	
HCV-RNA	(-)	
HBsAg	(-)	
HBcAb	(-)	
HAV-IgM	(-)	
HEV-IgA	(-)	
HTLV-1 Ab	(-)	
EBV-IgG	(+)	
EBV-IgM	(-)	
EBNA	(+)	
CMV-IgG	(+)	
CMV-IgM	(-)	
VZV-IgG	(+)	
VZV-IgM	(-)	
HSV-IgG	(+)	
HSV-IgM	(-)	
*H. pylori* Ab	(+)	

AST: aspartate aminotransferase; ALT: alanine aminotransferase; GGT: gamma-glutamyl transferase; ALP: alkaline phosphatase; BUN: urea nitrogen; eGFR: estimated glomerular filtration rate; CRP: C-reactive protein; TSH: thyroid stimulating hormone; FT4: free thyroxin 4; IgG: Immunoglobulin G; IgM: Immunoglobulin M; ANA: antinuclear antibody; AMA: antimitochondrial antibody; LKM1: antiliver/kidney microsome type 1; Ab: antibody; anti-SSA: anti-Sjögren's syndrome antigen A; anti-SSB: anti-Sjögren's syndrome antigen B; PAIgG: platelet-associated IgG; HLA: human leukocyte antigen; HCV: hepatitis C virus; HBsAg: hepatitis B surface antigen; HBcAb: hepatitis B core antibody; HAV: hepatitis A virus; HEV: hepatitis E virus; HTLV-1: human T-cell leukemia virus type 1; EBV: Epstein-Barr virus; CMV: cytomegalovirus; VZV: varicella zoster virus; HSV: herpes simplex virus; *H. pylori*: *Helicobacter pylori*.
